# Brain connectivity markers in degenerative cervical myelopathy patients with depression for predicting the prognosis following decompression surgery

**DOI:** 10.3389/fneur.2022.1003578

**Published:** 2022-10-24

**Authors:** Rui Zhao, Xu Chu, Yuqi Ge, Xing Guo, Yuan Xue

**Affiliations:** ^1^Department of Orthopedics Surgery, Tianjin Medical University General Hospital, Tianjin, China; ^2^Department of Orthopedics, Xi'an Jiaotong University Affiliated Honghui Hospital, Xi'an, China; ^3^Tianjin Key Laboratory of Spine and Spinal Cord, Tianjin Medical University General Hospital, Tianjin, China

**Keywords:** degenerative cervical myelopathy, inflammation, depression, functional connectivity, resting-state fMRI

## Abstract

**Objective:**

To determine if brain functional connectivity (FC) is associated with the prognosis in depressed degenerative cervical myelopathy patients (DCM) and to investigate the possible brain functional mechanism.

**Methods:**

Resting-state fMRI scans and peripheral blood cell counts from 33 depressed DCM patients, 33 age and gender-matched DCM patients without depression were analyzed. All patients were evaluated using Japanese Orthopedic Association score before and 6 weeks after decompression surgery. JOA recovery rate was calculated to assess the functional recovery for DCM patients. For each participant, seed-based functional connectivity maps based on sub-regions centered on the striatum were computed and compared between groups. Pearson correlations were performed to explore the relationships between clinical measures and brain alterations in depressed DCM patients. To further investigate the relationships between brain alterations and clinical measures in depressed DCM patients, mediation analyses were performed. Flow cytometry was also performed on the three of the 33 depressed DCM patients, and the results were analyzed.

**Results:**

In comparison to patients without depression, DCM patients exhibited lower FC between the dorsal caudate (dC) and the inferior frontal operculum, which is located in the dorsal lateral prefrontal cortex (dlPFC). In depressed DCM patients, the altered dC-dlPFC FC was associated with inflammation as determined by the neutrophils/lymphocyte's ratio and prognosis. Furthermore, the mediation analysis demonstrated that the dC-dlPFC FC mediated the effect of inflammation on prognosis. The outcomes of our three cases followed a similar pattern to these findings.

**Conclusion:**

In conclusion, our findings imply that inflammation slowed the functional recovery in depressed DCM patients through the striatal-frontal FC pathway.

## Introduction

Degenerative cervical myelopathy defined by acquired stenosis of the cervical spinal canal, causes progressive disability and paralysis due to chronic spinal cord compression and non-traumatic spinal cord injury (1), affecting more than 13% of individuals over the age of 65 worldwide (1, 2). Consequently, determining optimal treatment and clinical care strategies for DCM has been one of the top priorities for spine clinicians (3, 4). Over the past decades, the literature on DCM has extensively discussed the choice of treatment (5) (e.g., operative treatment or non-operative management), the timing of surgery (6–9), surgical approach (10) (e.g., anterior vs. posterior surgical procedures). However, in the management of DCM, psychological comorbidities such as depression have been largely overlooked. Previous epidemiologic studies have revealed that the prevalence of depression in DCM patients preoperatively is about 20% (11, 12); more importantly, psychiatric comorbidities, particularly depression, have been associated with poorer clinical outcomes in DCM patients (13–15). Given that depression is a significant and developing concern in DCM management, there was previously no valid, widely acknowledged approach for addressing depression in depressed DCM patients throughout the perioperative phase. This is partially due to the fact that the mechanisms underlying depression's influence on surgical outcomes are unknown (1).

In the past years, possible brain mechanisms have been put out by several neuroimaging studies. In these studies, it has been shown that the motor slowness in patients with depression is associated with decreased striatal-frontal functional connectivity (16–18). Moreover, further researches on this issue revealed that the decreased striatal-frontal functional connectivity is secondary to inflammation in these patients, because inflammation could be a cause of dopamine deficiency within reward-related brain regions (e.g., striatal area) and further aggravates the anhedonia and motor slowness in non-human primates (19–21). To further test these hypotheses, Miller et al. conducted resting-state functional connectivity in depressed patients and demonstrated that inflammation (i.e., measured by C-reactive protein; CRP) was strongly associated with motor slowing in patients with depression and that these effects of CRP on clinical symptoms were mediated by striatal-frontal functional connectivity (17). These findings imply that decreased striatal-frontal connectivity was associated with motor deficits in depressed patients with increased inflammation, indicating that the altered striatal-frontal connectivity could serve as a potential biomarker for predicting the emergence of motor deficits in patients with depression. However, it is still unknown whether such depression-related markers can be used to predict the postoperative motor function in DCM patients with depression. Exploring such markers could be crucial for understanding the mechanism underlying the poor outcomes in DCM patients with depression.

The current study used whole-brain blood oxygen level-dependent (BOLD) resting-state functional magnetic resonance imaging (rs-fMRI) (22–24) to determine whether increased inflammation (plasma neutrophils/lymphocytes ratio) is associated with altered striatal-frontal functional connectivity, which encodes reward processing and other goal-directed behaviors such as motor control (25–27). This technique has been demonstrated to be capable of elucidating a distinct pattern of functional connectivity between the whole-brain and each striatal sub-region. More importantly, this approach has demonstrated that the striatal-frontal functional connectivity identified by this approach is sensitive to dopaminergic pharmacological manipulation (27–29) and could be a potential druggable target. We hypothesized that the altered striatal-frontal functional connectivity would be associated with the prognosis in DCM patients with depression.

## Materials and methods

### Subjects

The ethical approval for this cross-sectional study was granted by the local institutional review board. All participants signed written informed consent before each procedure. Patients with degenerative cervical myelopathy (DCM) were recruited using the following criteria: (1) clear MRI evidence of myelopathy in the cervical spine (C3–C7); (2) clear clinical manifestations of myelopathy (e.g., sensorimotor extremity deficits, bladder/bowel dysfunction, gait disturbance, and others); (3) no history of cervical spinal surgery and agreement to undergo decompression surgery; (4) ability to complete fMRI studies; and (5) no stenosis of the extracranial vertebral artery or the carotid artery following Doppler ultrasound examination: (6) no clinical evidence or history of other neurological, psychiatric, ocular disease, or systemic diseases like hypertension and diabetes; and (7) no history of alcohol or substance abuse. The following criteria were used to recruit healthy subjects of similar age, gender, and education through advertisements (1) no evidence of spinal compression; (2) no other spinal or brain neurological disorders, or systemic disease; and (3) ability to complete fMRI studies. A total of 71 DCM patients and 50 Healthy Controls (HC) were recruited between 2015 and 2020.

### Clinical assessment

All DCM patients were carefully assessed by a senior orthopedic surgeon before the functional MRI (fMRI) scan. Each patient was evaluated using the Japanese Orthopedic Association (JOA) score, which is the most widely used scale for determining the severity of DCM disease in clinical practice. Subsequently, the same surgeon analyzed the JOA score (i.e., postoperative JOA score) 6 weeks after surgery to calculate the JOA recovery rate. When DCM patients were admitted to the hospital, the Beck Depression Inventory (i.e., edition v2.0) was assessed.

Between 7 and 8 a.m., fasting venous blood samples were obtained from 33 of 71 recruited DCM patients who were classified as having depression (i.e., with a BDI score above 20) for measurement of absolute blood cell counts (red blood cells, white blood cells, platelets, neutrophils, eosinophils, basophils, lymphocytes, and monocytes). Because the absolute counts of white blood cells, neutrophils, eosinophils, basophils, lymphocytes, monocytes as well as neutrophils/lymphocytes ratio (NLR) were considered to be associated with inflamed depression, subsequent analysis was limited to these blood metrics.

Additionally, three of the 33 depressed DCM patients volunteered to have their plasma interleukin-6 (IL6), plasma interleukin-2 (IL2), plasma interleukin-4 (IL4), and tumor necrosis factor-α (TNF-α) assessed using flow cytometry.

### fMRI data acquisition and preprocessing

#### Data acquisition

3T fMRI data were acquired using a MAGNETOM Prisma 3T MR scanner (Siemens, Erlangen, Germany) with a 64-channel phase-array head-neck coil. Sponge pads were performed on support the head for minimizing the head movement during the scan. All participants were clearly instructed to keep their eyes closed and remain awake, while avoiding specific and strong thoughts. Furthermore, the head motion of the functional scan of each participant was calculated and the participants whose head motion was not within defined motion thresholds (i.e., translational or rotational motion parameters <2 mm or 2°) were required to undergo the fMRI scan again to minimize the effect of head motion.

BOLD signals were collected using prototype simultaneous multi-slices gradient echo echo-planar imaging (EPI) sequence using the following parameters: echo time (TE) = 30 ms, repetition time (TR) = 800 ms, field of view (FOV) = 222 mm × 222 mm, matrix = 74 × 74, in-plane resolution = 3 mm × 3 mm, flip angle (FA) = 54 degree, slice thickness = 3 mm, gap = 0 mm, number of slices = 48, slice orientation = transversal, bandwidth = 1,690 Hz/pixel, PAT (parallel acquisition technique) mode, slice acceleration factor = 4, phase encoding acceleration factor = 2. Four hundred fifty images were taken in 6 min. A high-resolution 3D T1 structural image (two inversion contrast magnetization prepared rapid gradient echo sequence, MP2RAGE) was also acquired using the following parameters: TR/TE = 4,000 ms/3.41 ms, inversion times (TI1/TI2) = 700 ms/2,110 ms, FA1/FA2 = 4 degree/5 degree, matrix = 256 × 240, FOV = 256 mm × 240 mm, number of slices = 192, in-plane resolution = 1 mm × 1 mm, slice thickness = 1 mm, slice orientation = sagittal, total duration is 6 min, 42 s.

#### Data preprocessing

Functional MR data were preprocessed using the Data Processing Assistant for rs-fMRI (DPARSF; http://www.restfmri.net/forum/DPARSF) toolbox. The detailed preprocessing procedures were as follows: (1) The first 10 volumes of each functional scan were excluded due to the acclimatization to the scanning environment and magnetization stabilization; (2) Motion corrections were performed to remove the effect of head movement; (3) Functional images were co-registered to structural images and spatially normalized to the Montreal Neurological Institute template and each voxel was resampled to 3 × 3 × 3 mm^3^; (4) The liner-drift, Friston-24 parameters, the mean global signal, the white matter signal, and CSF signal were extracted as covariates and regressed out to minimize non-neural signals; (5) Subsequently, scrubbing for high motion timepoints was also performed; (6) Finally, a bandpass filter (0.01 ~ 0.08 Hz) was then applied to remove high-frequency noise effects; (7) resultant functional images were smoothed with an 8 mm full-width-half-maximum isotropic Gaussian kernel.

### Seed-to-whole-brain connectivity analysis

The seeds (i.e., region of interest, ROI) centered on ventral and dorsal striatal regions (i.e., a total of eight seeds), were selected based on Felger et al. (17). The detailed coordinates for ventral and dorsal striatal ROI are presented in Table 1. These ROIs were defined as regions exhibiting the maximal decrease in response to hedonic reward (gambling task) in subjects administered with the inflammatory cytokine interferon-alpha. The mean time-series within a specific ROI was extracted, and Pearson correlation coefficients between this time-series and the remaining voxel time-series were calculated. Subsequently, Fisher's *Z*-transformed were performed to acquire the *Z*-transformed brain maps and age, gender, and education years were regressed to rule out the possible effect of these confounders. The resultant maps were used for further analysis.

**Table 1:** Coordinates for ventral and dorsal striatal regions of interest.

**Region of interests**	**Coordinates**		
Inferior ventral striatum (iVS)	±14	8	−9
Ventral rostral putamen (vrP)	±20	12	−3
Dorsal caudal putamen (dcP)	±28	1	3
Dorsal caudate (dC)	±13	15	9

The negative X coordinate means left, the positive X coordinate means right.

#### Analysis 1: Correlation analysis for clinical assessments in depressed DCM patients

Pearson correlation coefficients were calculated among preoperative JOA score, JOA recovery rate, and the absolute counts of white blood cells, neutrophils, eosinophils, basophils, lymphocytes, monocytes, as well as the NLR in DCM patients with depression.

#### Analysis 2: Seed-to-whole-brain connectivity analysis

It is important to note that the current analysis has used non-depressed DCM patients as a control group in comparison to depressed DCM patients. In this case, when comparing the brain alterations and prognosis between these two groups, we can isolate the effect of depression on functional connectivity and JOA recovery rate in DCM patients from other factors such as preoperative disease severity (i.e., differences in preoperative JOA score between depressed DCM patients and healthy controls). Therefore, we divided the DCM patients into depression group and non-depression group and optimally matched the preoperative JOA scores between two groups, paired-*t* test was performed between group to further explore the baseline differences in JOA score between two subgroups. That is, for a given subject in the depression group, we select a DCM patient without depression who's preoperative JOA score is closest to that of the given subject. The JOA recovery rate was also compared between two groups (e.g., depressed DCM patients and non-depressed DCM patients).

Subsequently, a voxel-wise two-sample *t*-test was performed to reveal the functional connectivity differences within the gray matter masks using SPM12 (http://www.fil.ion.ucl.ac.uk/spm). Voxel-level *p*-value = < 0.001 (significance threshold) was corrected for multiple comparisons using family-wise error correction at the cluster level, resulting in a corrected *p* = <0.05. Clusters that showed significant differences between groups were selected and mean FC value of each depressed DCM patients were then extracted to correlate with clinical assessments.

#### Analysis 3: Mediation analysis

A bootstrapped mediation analysis was performed to assess the mediatory relationship between NLR, JOA recovery rate, and seed-based functional connectivity. The PROCESS macro (www.processmarcro.org, version 2.16.3) in SPSS (IBM, version 23.0.0) was used with 5,000 bootstrap samples, to identify 95% confidence intervals for model components. The mediation analysis was conducted to determine whether there was a significant difference between the total effect (path c) and the direct effect (path *c*′) when the mediator (*M*) was included (Figure 4). We investigated the association among NLR, JOA recovery rate and dC-lIFO functional connectivity. We constructed the model using the dC-lIFO functional connectivity as the mediator, NLR as the independent variable and JOA recovery rate as the outcome. We also constructed a model using dC-lIFO functional connectivity as the mediator, JOA recovery rate as the independent variable and NLR as the outcome to validate our results. We hypothesized that the first model would be significant and the second one would not. When the bootstrapped upper and lower 95% confidence intervals (CIs) did not include zero, there was a significant mediation.

#### Analysis 4: Case reports for DCM patients who underwent flow cytometry assessment

In this study, three of the 33 depressed DCM patients volunteered to have their plasma interleukin-2 (IL2), plasma interleukin-4 (IL4), and tumor necrosis factor-α (TNF-α) assessed using flow cytometry. This study aimed to explore the relationship between inflammatory proteins and prognosis in these patients. The ldC-lIFO, JOA recovery rate, IL2, IL4, and TNF-α were illustrated and compared among these patients.

## Results

### Demographic data and clinical assessments

The demographic data and the clinical assessments of all participants are summarized in Table 2. There were no significant inter-group differences with regard to age, gender, or years of education (*p* = <0.05).

**Table 2:** Demographic data of the two groups.

	**DCM (*n* = 33)**	**HC (*n* = 33)**	***p*-value**
Age (years)	55.2 ± 7.28	55.1 ± 6.41	0.75
Gender (F/M)	16/17	16/17	1
Education (years)	11.9 ± 3.21	12.1 ± 3.51	0.41
JOA	10.9 ± 1.75		
JOA recovery rate	64.7 ± 15.71		
BDI	24.31 ± 3.17		

DCM, degenerative cervical myelopathy; HC, healthy controls; JOA, Japanese Orthopedic Association; BDI, baker depression inventory.

#### Analysis 1: The absolute blood cell counts of neutrophils, lymphocytes, NLR are associated with surgical outcome in depressed DCM patients

**Figure 1:**
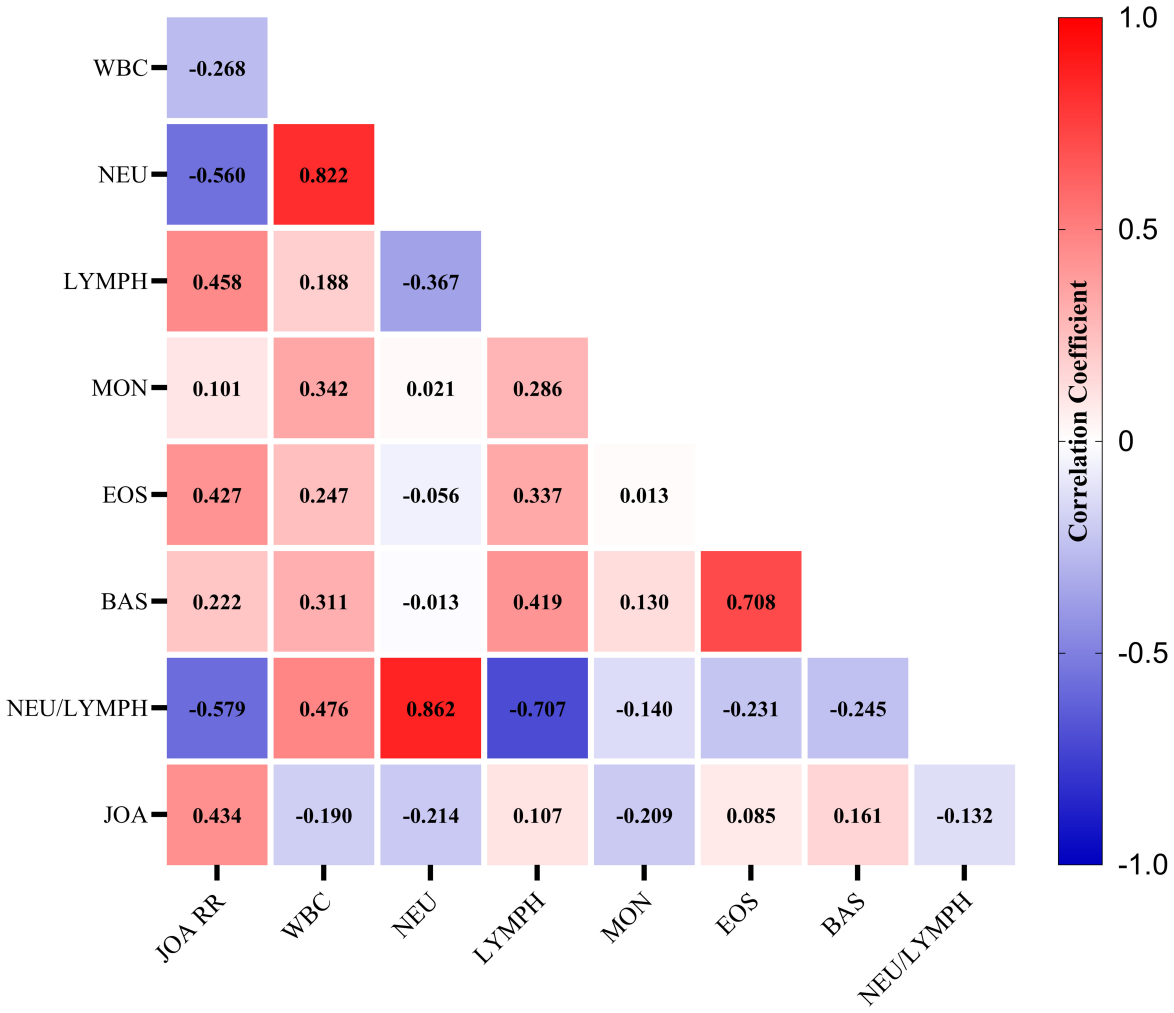
The heatmap for the correlations among clinical measures. The correlation coefficients were given in the center of each square. WBC, white blood cell counts; NEU, neutrophils count; LYMPH, lymphocyte counts; MON, monocyte counts; EOS, eosinophils count; BAS, basophils count; NEU/LYMPH, neutrophils/lymphocyte ratio.

We detected a significant correlation between the JOA recovery rate and neutrophils counts (*R* = −0.56, *p* < 0.001), lymphocytes count (*R* = 0.46, *p* = 0.01), and NLR (*R* = −0.58, *p* < 0.001). Furthermore, we detected a significant positive correlation between preoperative JOA score and JOA recovery rate (*R* = 0.43, *p* = 0.02). This finding was consistent with previous research demonstrating a strong correlation between preoperative neurological function and the prognosis of DCM following decompression surgery (Figure 1).

#### Analysis 2: Altered caudate-frontal functional connectivity is observed in DCM patients with depression

**Figure 2:**
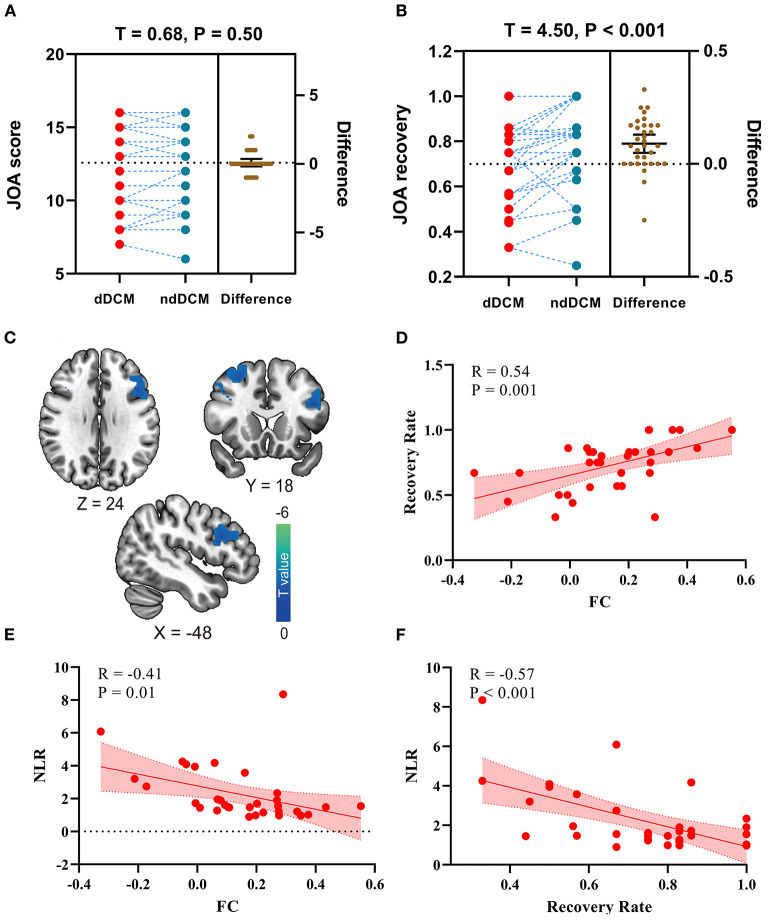
**(A)** The preoperative Japanese Orthopedic Association (JOA) score differences in depressed DCM (dDCM) patients and non-depressed DCM (ndDCM) patients; **(B)** The Japanese Orthopedic Association (JOA) recovery rate differences in depressed DCM (dDCM) patients and non-depressed DCM (ndDCM) patients; **(C)** Voxel-wise functional connectivity differences between depressed DCM patients and non-depressed DCM patients; **(D)** The correlation between functional connectivity (FC) and JOA recovery rate; **(E)** The correlation between FC (between dorsal caudate - inferior frontal operculum) and neutrophils/lymphocyte's ratio (NLR); **(F)** The correlation between NLR and JOA recovery rate.

We found that after controlling the preoperative JOA score, there is no significant differences in preoperative JOA scores between depressed and non-depressed JOA score (*t* = 0.68, *p* = 0.50; Figure 2A). Further, relative to non-depressed DCM patients, depressed DCM patients exhibited significant lower JOA recovery rate (*t* = 4.50, *p* < 0.001; Figure 2B).

In this study, eight seeds centered on ventral and dorsal striatal regions were used. In comparison to non-depressed DCM patients, depressed DCM patients exhibited significantly lower functional connectivity (FC) between the left dorsal caudate (ldC) and the left inferior frontal operculum (lIFO). The detailed information of the resulting clusters is presented in Table 3 and Figure 2C depicts their spatial distribution. In this study, no significant inter-group differences in other seed-based functional connectivity were observed.

**Table 3:** The detailed information for between-group functional connectivity differences (dorsal caudate as seed).

**Brain regions**	**Brodmann area**		**MNI coordinates**		**Peak intensity**	**Voxel size**
Left inferior frontal gyrus	BA 48, 46, 6	−54	15	30	−3.51	193

Additionally, a significant negative correlation was observed between the ldC-lIFO FC and the JOA recovery rate (*R* = 0.54, *p* < 0.001, Figure 2D), while a significant correlation was observed between the ldC-lIFO FC and NLR (*R* = −0.41, *p* = 0.01, Figure 2E) in depressed DCM patients. Figure 2F, depicts a scatter plot for the significant association between NLR and JOA recovery rate (*R* = −0.57, *p* < 0.001).

#### Analysis 3: The effect of NLR on JOA recovery rate is mediated by the ldC-lIFO functional connectivity

**Figure 3:**
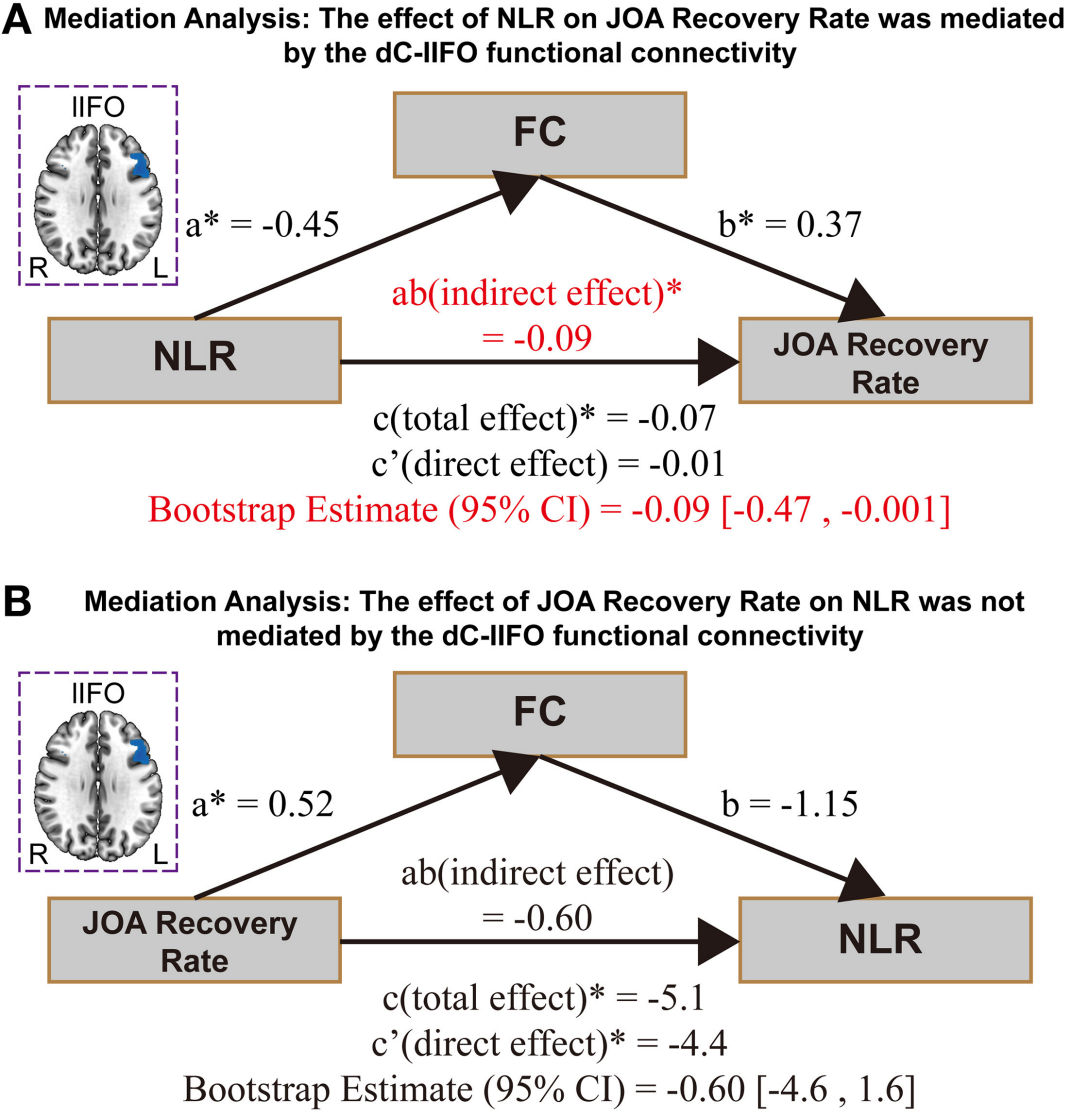
The results for mediation analysis. **(A)** The effect of NLR on JOA recovery rate. **(B)** The effect of JOA recovery rate on NLR. NEU/LYMPH, neutrophils/lymphocyte ratio; JOA, Japanese Orthopedic Association; dC, dorsal caudate; IFO, inferior frontal operculum.

The effect of NLR on the JOA recovery rate was mediated by the ldC-lIFO functional connectivity [direct effect = −0.01, *p* > 0.05; indirect effect = −0.09, 95% confidence interval: (−0.47, −0.001), Figure 3A]. Please note that the top panel of Figure 3 depicts the lIFO region used in the mediation analysis. In contrast, the effect of JOA recovery rate on NLR was not mediated by the ldC-lIFO functional connectivity [direct effect = −4.4, *p* < 0.05; indirect effect = −0.60, 95% confidence interval: (−4.6, 1.6), Figure 3 B].

#### Analysis 4: Depressed DCM patients with a higher level of inflammatory cytokine exhibit decreased dC-lIFO functional connectivity along with poorer prognosis following decompression surgery

**Figure 4:**
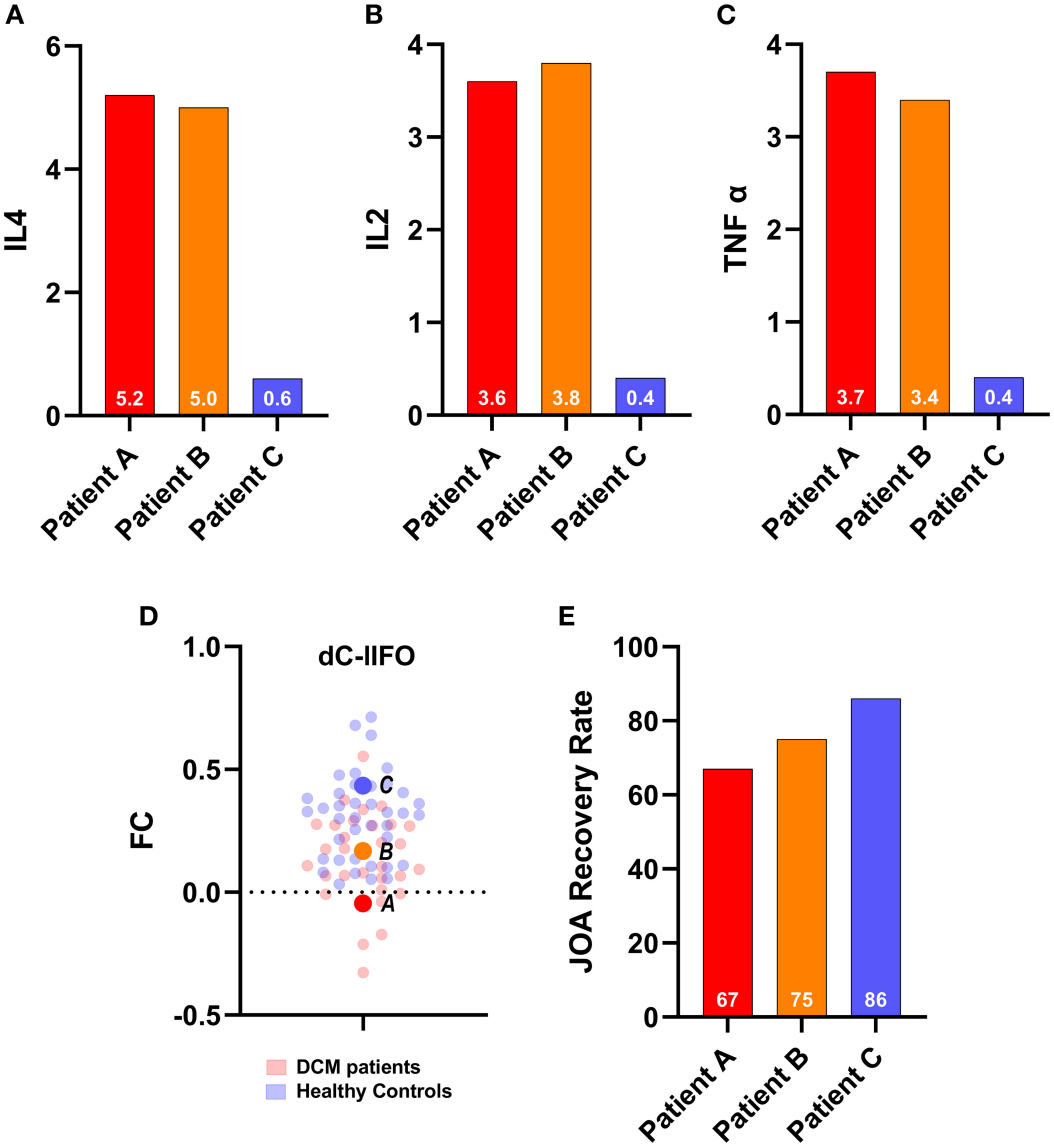
Cases report. Upper panel: The inflammatory cytokines of the three of 33 depressed degenerative cervical myelopathy patients. **(A)** IL4 for three patients, **(B)** IL2 for three patients, **(C)** TNF alpha for three patients, **(D)** The FC for three patients and their relative position among DCM patients and healthy controls, and **(E)** JOA recovery rate for three patients. JOA, Japanese Orthopedic Association; dC, dorsal caudate; IFO, inferior frontal operculum; FC, functional connectivity; DCM, degenerative cervical myelopathy.

In this study, three of the 33 DCM patients volunteered to undergo flow cytometry assessment of plasma interleukin-2 (IL2), plasma interleukin-4 (IL4), and tumor necrosis factor-α (TNF-α). Patient A exhibited a plasma IL4 concentration of 5.2 pg/ml, a plasma IL2 concentration of 3.6 pg/ml, a plasma TNF-α concentration of 3.7 pg/ml, and a 67% JOA recovery rate. Patient B exhibited a plasma IL4 concentration of 5.0 pg/ml, a plasma IL2 concentration of 3.8 pg/ml, a plasma TNF-α concentration of 3.4 pg/ml, and a 75% JOA recovery rate. Patient C exhibited a plasma IL4 concentration of 0.6 pg/ml, a plasma IL2 concentration of 0.4 pg/ml, a plasma TNF-α concentration of 0.7 pg/ml, and an 86% JOA recovery rate (Figure 4). We discovered a trend in this study that a higher level of inflammatory cytokine was associated with a poor prognosis in depressed DCM patients. This finding is consistent with those in previous results from analysis 3.

## Discussion

In this study, four significant findings emerged: (1) Inflammation as measured by NLR was associated with prognosis (i.e., as measured by JOA recovery rate) in depressed DCM patients following decompression surgery; (2) Relative to non-depressed DCM patients, DCM patients with depression exhibited altered caudate-frontal functional connectivity, and these alterations were associated with the prognosis of DCM; (3) The effect of inflammation on prognosis was mediated by the ldC-lIFO functional connectivity in DCM patients; (4) Our cases also indicated that a higher level of inflammatory cytokine was associated with a poorer prognosis in depressed DCM patients following decompression surgery.

### Altered striatal-frontal functional connectivity could be used as a potential biomarker for prognosis prediction in depressed DCM patients

In comparison to non-depressed DCM patients, depressed DCM patients had significantly decreased functional connectivity between the left dorsal caudate (ldC) and the left inferior frontal operculum (lIFO); and had significant lower JOA recovery rate. Increased inflammation as measured by the plasma NLR was found to be negatively correlated with ldC-lIFO functional connectivity, and the altered ldC-lIFO functional connectivity was positively correlated with the JOA recovery rate in depressed DCM patients. The lIFO regions, which are identified by seed-to-whole-brain functional connectivity analysis, and are located in the dorsal lateral prefrontal cortex (dlPFC) (30) have been demonstrated to alter mood and behavior in depression patients (31). Additionally, it has been demonstrated that individual differences in the severity of anhedonia/motivation symptoms were significantly associated with reduced sustained dlPFC activation (16). In schizophrenia patients, significantly reduced functional connectivity between the left dorsal caudate (dC) and bilateral dlPFC (e.g., located at the Brodmann Area 46) was identified and the altered dC-dlPFC functional connectivity was associated with the fine motor tasks that are sensitive to psychomotor speed (32). These findings established the critical involvement of dC-dlPFC functional connectivity in encoding motor function. Additionally, a recent study conducted by Miller et al. demonstrated an association between dC-dlPFC functional connectivity (e.g., The dlPFC were mainly located at the BA 6) and motor slowing in patients with depression (17). Our current findings from analysis 2 corroborated previous findings indicating that dC-dlPFC functional connectivity is associated with inflammation and motor function. Despite the slight differences in the location of dlPFC between our findings and previous studies, a significant part of our findings overlapped with those of previous studies (e.g., the lIFO we observed partly located at the BA6 and BA46). Our findings provided preliminary evidence that the altered striatal-frontal functional connectivity could be used as a potential biomarker for predicting the functional recovery following decompression surgery in depressed DCM patients.

### Inflammation has an effect on postoperative sensorimotor function recovery in depressed DCM patients *via* the dC-lIFO functional connectivity

In this study, we observed a significant correlation between inflammation as measured by NLR and surgical outcomes in depressed DCM patients. In both mood disorders (33) and spinal cord injury, the peripheral NLR was considered to reflect the activation of inflammatory processes (34). Zhao et al. (34) recently demonstrated that a higher peripheral blood neutrophil/lymphocyte ratio upon admission to hospital is associated with a poor outcome in patients with acute cervical spinal cord injury. This could be explained by the disruption of the blood–spinal cord barrier (BSCB) (35, 36), in which the integrity of the BSCB was impaired throughout the traumatic process, hence aggravating inflammation. As a result, inflammation-induced neuronal loss and demyelination exacerbate the spinal cord injury. Additionally, the such pathological process has also been identified in DCM (37). Compression of the spinal cord is considered to trigger endothelial cells dysfunction, which is critical for the integrity of the BSCB (38), and inflammatory cytokines may enter the central nervous system *via* disrupted BSCB.

Additionally, we identified a significant correlation between inflammation as measured by NLR and dC-lIFO functional connectivity in DCM patients with depression. Several studies on inflammation and depression have provided a plausible explanation. It has been demonstrated that impaired functional connectivity between dorsal caudate and dlPFC is strongly correlated with inflammation as measured by C-reactive protein in patients with major depressive disorder. More importantly, it has been demonstrated that the dC-dLPFC functional connectivity mediates the association between inflammation and behavior (e.g., motor slowing) (17). A plausible explanation for this pathway was suggested by translational neuroimaging and *in vivo* micro-dialysis studies (21). In these studies, researchers found that chronic administration of inflammatory cytokines decreased striatal dopamine release, which had a direct effect on the reward-related circuitry and resulted in anhedonia and motor slowness in monkeys (21). Additionally, the levodopa restored the decreased symptoms. These findings revealed that inflammation disrupted the mesocorticolimbic dopamine innervation, which is located in the dorsal striatal area and plays important roles in reward, motivation, learning, memory, and movement (39), leading to altered functional connectivity between the dlPFC and dC. The altered striatal-frontal functional connectivity was further associated with the motor slowness in patients with depression.

### Depressed DCM patients with a higher level of inflammatory cytokine exhibit decreased dC-dlPFC functional connectivity along with poorer prognosis following decompression surgery

In analyses 2 and 3, we used NLR as a measure to assess inflammation in DCM patients with depression. Even though the NLR is a reliable metric for determining the inflammation level and immune system activation, it is not a direct reflection of inflammation. To address this gap, we performed flow cytometry on three of the 33 depressed DCM patients. We quantified inflammatory cytokines such as IL-2, IL4, and TNF-α. Additionally, we identified a trend that patients with a higher level of inflammation exhibited decreased dC-dlPFC functional connectivity, which was associated with a poorer prognosis. This finding further supported our previous observation.

### Limitations

There are several limitations to this study. First, the main limitation is that we used NLR as an indicator for inflammation in depressed DCM patients. NLR is not a direct measure of inflammation, in comparison to C-reactive protein and other inflammatory cytokines. It reflects the inflammatory process by estimating the level of immune system activation. Although our case reports corroborated our findings, additional investigations using CRP or flow cytometry are required to further validate our findings. Second, all of the DCM patients included in this study followed strict conservative treatment (e.g., physical therapy, a non-steroidal anti-inflammatory drug, muscle relaxants, and neurotrophic drugs). This may affect our results to some extent. Therefore, future studies with DCM patients who are not on medication or who have a washout period from medication are needed to confirm our findings. Further, our current study applied BDI for screening DCM patients with depression and thus could not correlate the severity of depression with any brain alteration. The association between depression symptom and our observed ldC-lIFO need future study for validation. Finally, postoperative fMRI data was not collected due to the possibility of artifact and heating due to surgical implants. Even though it appears to be safe and other studies have collected data on postoperative fMRI data, the majority of our patients declined to cooperate after we informed them of potential harm (e.g., loss of surgical implants) associated with postoperative fMRI.

## Conclusion

In conclusion, our findings suggest that inflammation may impair corticostriatal connectivity and further affect recovery from decompression surgery in patients with degenerative cervical myelopathy who also suffer from depression. This finding might add future pharmacological studies aimed at developing treatment strategies for depressed patients with degenerative cervical myelopathy.

## Data availability statement

The raw data supporting the conclusions of this article will be made available by the authors, without undue reservation.

## Ethics statement

The studies involving human participants were reviewed and approved by Tianjin Medical University General Hospital. The patients/participants provided their written informed consent to participate in this study.

## Author contributions

YX: designed the study. RZ: analyzed the data and wrote the draft. YG and XC: collected the data. XG: analyzed the data. XC: validated the results and revised the manuscript. All authors contributed to the article and approved the submitted version.

## Funding

This study has received funding by National Natural Science Foundation of China (grant numbers: 81871124, 81471403, 30973024, and 61773223).

## Conflict of interest

The authors declare that the research was conducted in the absence of any commercial or financial relationships that could be construed as a potential conflict of interest.

## Publisher's note

All claims expressed in this article are solely those of the authors and do not necessarily represent those of their affiliated organizations, or those of the publisher, the editors and the reviewers. Any product that may be evaluated in this article, or claim that may be made by its manufacturer, is not guaranteed or endorsed by the publisher.
